# Dynamic econometric analysis on influencing factors of production efficiency in construction industry of Guangxi province in China

**DOI:** 10.1038/s41598-022-22374-y

**Published:** 2022-10-20

**Authors:** Ting Ouyang, Fengtao Liu, Bingzhang Huang

**Affiliations:** Liuzhou Institute of Technology, Liuzhou, 545616 Guangxi China

**Keywords:** Civil engineering, Statistics

## Abstract

China's construction industry has assumed an important role in China's urbanization process, improving China's urban landscape and the level of national production and living facilities, but the productivity of the construction industry in some regions of China is still at a relatively low level. Taking the construction industry in Guangxi province in southwest China as an example, this paper analyzes the relevant indexes affecting the total factor productivity level of the regional construction industry and composes the statistical relationships among the indexes using dynamic measurement methods, and obtains that: (1) The number of employees, enterprises, labor productivity and construction profit have positive influence on the total factor productivity of Guangxi construction industry, but the improvement of regional construction gross product does not drive the improvement of technical equipment rate; (2) There is a dynamic equilibrium relationship between input and output indicators of total factor productivity of Guangxi construction industry, and the positive driving effect of output indicators on input indicators is not obvious; the influence of input indicators on output indicators is greater, and the positive influence is more. Accordingly, this paper also puts forward corresponding suggestions to promote the technical production level of Guangxi's construction industry.

## Introduction

The construction industry, as one of the pillar industries of China's national economy, has contributed greatly to the improvement of the gross national product over the years. At the same time, the construction industry, as an important way to improve urban infrastructure, makes a large amount of social production investment and is also an important element of China's infrastructure projects, thus the influence of the construction industry on the entire national economy is worthy of in-depth study^1^. As an underdeveloped region in China, Guangxi's gross national product ranks low among the 31 provinces and cities in China (fluctuate after 20th in 31 provinces and cities of China for many years) , and with the intensive and deep communication and trade with ASEAN, and after receiving a series of domestic and international policy support, Guangxi's urban development has greater potential, and the construction industry plays an important role in Guangxi's economic construction, but because the overall production level of Guangxi's construction industry is not high, the production efficiency and Technical innovation and other aspects still need to be improved.

Considering the development status of Guangxi construction industry, in order to correspond to the call of national policy and improve the development speed of Guangxi construction industry, we should pay full attention to the improvement of production level of construction industry, improve the production efficiency and explore the endogenous mechanism of relevant influencing factors. The economic development of Guangxi is lower than that of other provinces in China. The important role of the construction industry in the local economy has been proved many times in the many construction industry research of provinces in China^2,3^. Based on the current development status of Guangxi, how about the total factor production level of Guangxi construction industry, whether there is statistical correlation between indicators, and whether there is a mutual influence between indicators. The purpose of the research is to evaluate the total factor productivity(TFP) of the construction industry in Guangxi province, and finding out the relationship among the indicators of TFP, so as to provide directions and ideas for the formulation of relevant policies to improve the level of the construction industry in Guangxi province of China.

**Figure 1:**
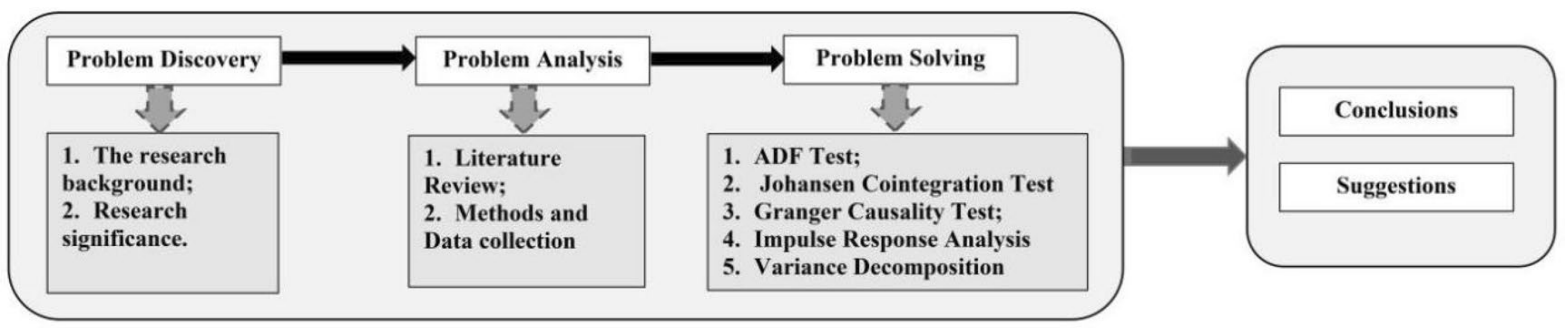
Research Path and Structure.

This research follows the research idea of problem discovery to problem analysis and then problem solving, and the research pathway is shown in Fig. 1:

The research has dual significance. The theoretical significance lies in enriching the research results on the total factor productivity (TFP) of China's construction industry and filling the research gap on the construction industry productivity of Guangxi province, which is a underdeveloped region in southwest China. The practical significance lies in sorting out the development characteristics of Guangxi construction industry and the dynamic measurement relationship between the indicators of TFP, which provides the direction for the key factors of sustainable development of Guangxi construction industry in the future.

## Literature review

In the 1990s, the British economist Farrell explained economic efficiency on the basis of the theory of Pareto optimality, suggesting that efficiency refers to the ratio of inputs to outputs in the production process, among which the concept of "total factor productivity" was proposed by Stigler for the construction industry. Subsequently, on the basis of this theory, many scholars have conducted diverse studies on the productivity of various industries^4,5^ and have continuously improved this concept based on parametric and non-parametric approaches^6,7^.

K.W. Cha^8^ started to focus on labor productivity in the construction industry as early as 1988, and in order to avoid one-sided labor productivity studies, he used a combination of construction cost index and price index to measure the productivity of the construction industry in Hong Kong from another perspective. In 2003, Mao Zhi et al. analyzed and calculated the relevant indicators of the construction industry in Singapore and used Jorgenson's method to measure the data during the fifteen years from 1984 to 1998 to improve the measurement of total factor productivity in the construction industry^9^. Asheim used the analysis of data related to Norwegian construction firms and found that the average efficiency level of firms in their statistical sample was high, where the factors that had a greater impact on efficiency were high wages, high working hours, etc^6^ . Yousong W et,al. used DEA calculations to analyze the productivity level of the construction industry in Hong Kong during 1981–1994 and found that Hong Kong's construction industry grew steadily during this 14-year period, with larger firms and standardized management, and higher productivity in firms with lower subcontracting rates^10^. Xueqing Wang and Shuguo Zhou et al. conducted an in-depth study on the efficiency and measurement of the construction industry, using a DEA model for the first time, with a three-stage differentiation of the analysis process, and an empirical analysis using economic indicators related to the construction industry in 30 provinces and cities across China in 2008, and found that by improving the level of technicalization and project management in the construction industry, the optimal allocation of resources could be better achieved, thus promoting the efficiency of the construction industry. The empirical analysis found that by improving the level of technicalization and project management in the construction industry, the optimal allocation of resources could be better achieved, thus promoting the efficiency of the construction industry^11,12^. Subsequently, Li Zhongfu et al. conducted a Malmquist index trend of the indicators related to the construction industry in China from 1996 to 2005 by DEA method, and found that the construction industry in China has changed from rough to intensive, and technological progress has a significant role in improving the productivity of the construction industry^13–16^.

From the literature review, many scholars focus on evaluation of the productivity of the construction based on the different panel data and index, including setting different index of each region at home and abroad, but research of the relationship between factors in TFP index,or the factors affect each other are insufficient^17–23^.Therefore, the innovation of this study lies in the micro analysis and found the dynamic measurement relationship between various factors of the construction industry production efficiency evaluation system, which provides a basis for the future development of the correlation analysis in construction industry production factors, and also provides the control measures of improving the construction industry production efficiency.

## Methods and data

### Parameter correlation proof

At this stage, the explanatory variables are screened, which is set as the simplest multiple linear regression model^24^. The general form of multiple linear regression model is:

1 $$Y_{i} = \beta_{0} + \beta_{1} X_{1i} + \beta_{2} X_{2i} + \cdots + \beta_{k} X_{ki} + u_{i} (i = 1,2, \ldots ,n)$$

In the above equation, k represents the number of explanatory variables, i.e., the number of variables to be filtered out in this paper, while β_0_, β_1_…β_k_ represent the unknown parameters, also known as Regression Coefficient^25^, in econometrics, β_0_ becomes the Intercept term, while β_1_…β_k_ is called the Slope Coefficients.

The part of $Y_{i} = \beta_{0} + \beta_{1} X_{1i} + \beta_{2} X_{2i} + \cdots + \beta_{k} X_{ki}$ in the above equation is called the systematic part or the deterministic part, while ui is a random variable used to represent the deviation, also called Stochastic Disturbance, which is called the stochastic part or the non-systematic part. Such a linear model indicates that the factors affecting the explanatory variable Y, except for the explanatory variable X_ji_, which is already included in the overall regression model, and other factors affecting Y but not included in the model are uniformly represented by the random disturbance term u_i_. The inclusion of random disturbance term in the model can firstly include objectively existing factors that have not been considered or data that cannot be obtained into the calculation, secondly allow model setting errors and data measurement errors, and finally avoid random influence among variables in the model. so as setting u_i_ as random disturbance term has a very important significance and is indispensable in the process of performing model construction, which has an important role in the calculation and testing of the model and the empirical validity of the model^26,27^.

### Index selection and data

The selection of indicators follows the following principles:

1. Validity and objectivity
  This is the fundamental principle of index selection, which should be closely related to the TFP of Guangxi province, but at the same time, the mutual substitution of indicators should be avoided.

(2) Comprehensive
  TFP involves input and output indicators, which has different dimensions and different types. In order to avoid the absence of relevant indicators or measurement errors, the selection of indicators should be guided by comprehensive principles.

(3) Feasibility and scalability
  In order to ensure that the model can be used for empirical testing, the indicators must be feasible. The data which are easy to obtain and have accurate data are preferred in the selection of indicators.

According to the basic idea of input–output screening the indicators for calculation. In order to study the influencing factors of the TFP of the construction industry in Guangxi, considerating the limitations of Guangxi statistical data , this research selects the data of six indicators of gross output value, number of employees, number of enterprises, labor productivity, technical equipment rate and total profit of Guangxi construction industry were obtained by referring to relevant literature^28–34^ , as shown in Table 1:

**Table 1:** Total factor productivity index system of Guangxi construction industry.

Evaluation object	Tier 1 indicators	Tier 2 indicators	Indicator components
Total factor productivity of Guangxi construction industry	Input index	Human input resource input	Employees X_1_
			number of enterprises X_2_
			technical equipment rate X_3_
	Output index	Technical output	Labor productivity X_4_
		Economic output	Total profit of construction industry X_5_
			Gross output value of construction industry X_6_

The original data of the above indicators from 2004 to 2020 are shown in Table 2 after consulting the Guangxi statistical yearbook:

**Table 2:** Original data of relevant indicators of total factor productivity of Guangxi construction industry from 2004 to 2020.

Year	Employees (10,000)	Number of enterprises	Labor productivity (yuan/person)	Technic-al equipment rate (yuan/person)	Total profit (10,000 yuan)	Gross output value of construction industry (100 million yuan)
2004	34.00	1017	102,340	7866	49,089	333.59
2005	43.00	1047	125,917	8843	79,337	425.21
2006	45.10	1048	149,494	7439	104,970	512.83
2007	47.80	1087	166,742	7486	130,578	612.74
2008	43.30	1123	219,028	9878	150,968	753.21
2009	53.40	1151	220,694	7962	197,443	934.38
2010	59.06	1160	257,132	6960	258,743	1222.31
2011	59.48	1174	315,302	7103	262,368	1,553.07
2012	67.03	1258	366,174	6745	342,674	1867.06
2013	76.50	1245	385,924	5280	444,218	2289.88
2014	77.92	1163	343,180	5307	506,542	2608.91
2015	85.67	1152	344,720	5292	543,178	2953.42
2016	113.29	1203	373,289	3726	681,397	3434.33
2017	139.29	1326	368,255	3139	119,006	4209.72
2018	122.37	1481	404,019	2686	162,108	4401.25
2019	141.97	1703	484,409	2419	1,014,041	5407.31
2020	92.00	1913	402,603	2365	872,224	5853.24

The labor productivity is calculated according to the gross output value.

For the convenience of statistics and further calculation, descriptive statistics are made on the above raw data, and the results are shown in Table 3:

**Table 3:** Descriptive statistics of Guangxi construction industry from 2004 to 2020.

	Mean	Median	Max	Min	Std.Dev	Skewness	Kurtosis	Jarque–Bera	Prob
Gross output value of construction industry	2316.03	1867.06	5853.24	333.59	1798.17	0.64	2.16	1.66	0.44
Employees (10,000)	76.54	67.03	141.97	34.00	34.52	0.70	2.24	1.80	0.41
Number of enterprises	1250.06	1163	1913	1017	240.58	1.65	4.87	10.23	0.005
Labor productivity (Yuan/person)	295,836.6	343,180	484,409	102,340	113,307.5	− 0.31	1.91	1.12	0.57
Technical equipment rate (Yuan/person)	5911.53	6745	9878	2365	2360.72	-0.19	1.85	1.03	0.60
Total profit (10,000 yuan)	348,169.6	258,743	1,014,041	49,089	288,718.8	1.04	2.98	3.04	0.22

**Figure 2:**
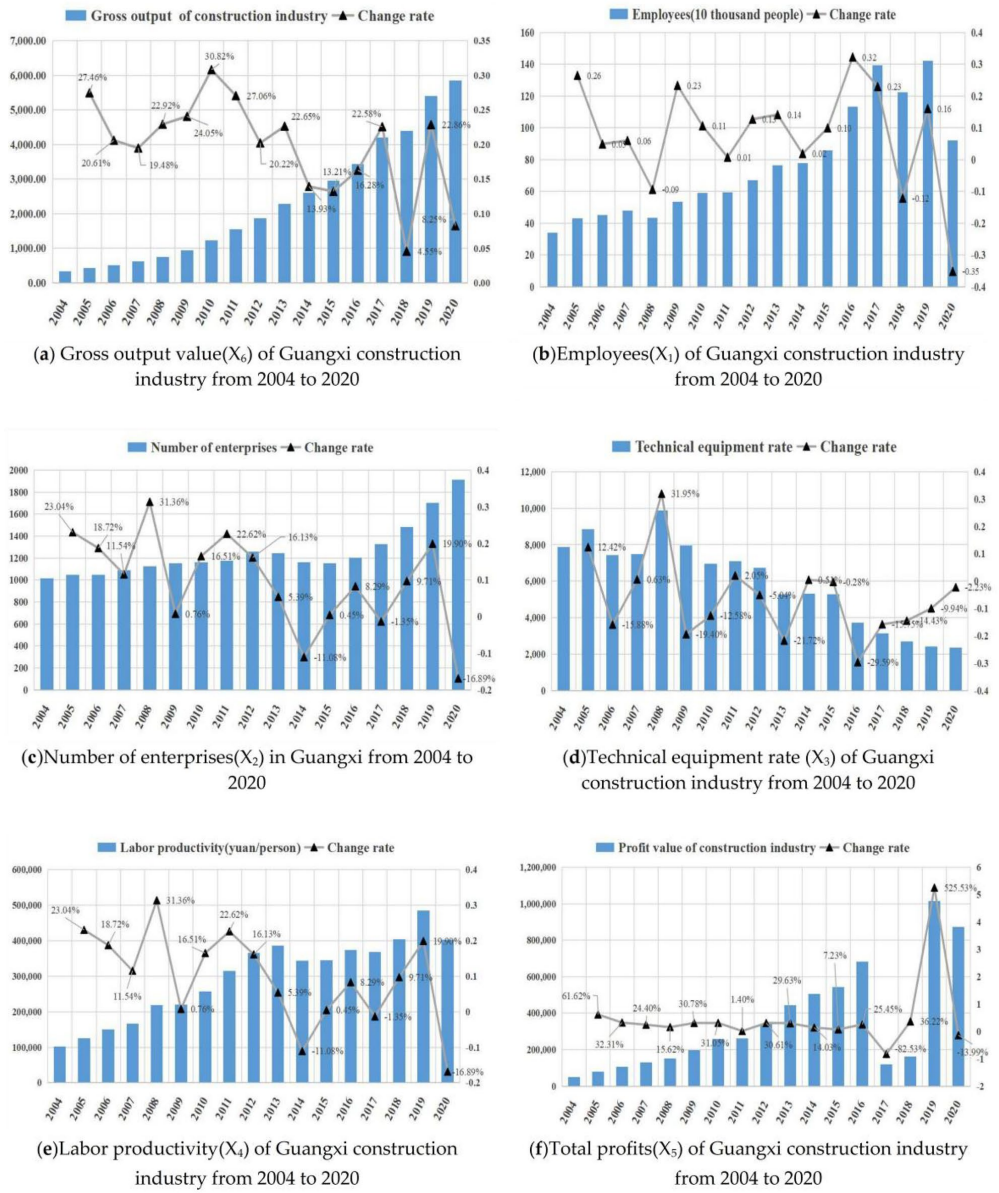
Various factors of Total factor productivity trend from 2004 to 2020:(**a**) Gross output value(X_6_) of Guangxi construction industry from 2004 to 2020;(**b**)Employees(X_1_) of Guangxi construction industry from 2004 to 2020;(**c**)Number of construction enterprises(X_2_) in Guangxi from 2004 to 2020;(**d**)Technical equipment rate (X_3_) of Guangxi construction industry from 2004 to 2020;(**e**)Labor productivity(X_4_) of Guangxi construction industry from 2004 to 2020;(**f**)Total profits(X_5_) of Guangxi construction industry from 2004 to 2020.

In accordance with the data content of Table 1 and Table 2, we can get Fig. 2a–f to show the trend of gross output value, number of employees, number of enterprises, labor productivity, technical equipment rate and total profit of Guangxi construction industry from 2004 to 2020 respectively.

Figure 2a shows the development of the gross value of construction industry in Guangxi province from 2004 to 2020, and it can be seen that the construction industry in Guangxi has shown a development trend in general in these 17 years, and the gross value of construction industry has increased year by year, but the growth rate fluctuates frequently, and it is also found in Fig. 2b,c,e that the employees, the number of enterprises and the labor productivity of the construction industry in Guangxi have increased in general from 2004 to 2020 with fluctuating, which has some similarity with the change of Guangxi construction industry's GDP. While the technical equipment rate of Guangxi construction industry in Fig. 2d decreases year by year, which is opposite to the development trend of Guangxi construction gross product, indicating that there may be a negative correlation; while the profit of Guangxi construction industry in Fig. 2f has a large change in 2017 and 2018, except for 2019 when it reached a higher peak value, the overall trend has some similarity with the development of Guangxi construction gross product, but the overall change is more stable. Therefore, further verification of the correlation with the gross construction product index is needed.

The purpose of this study is to analyze the influence of various factors on the development of the construction industry in Guangxi, and the gross value of construction industry in Guangxi as a commonly used core economic indicator, and set the gross value of construction industry as the explanatory variable in this study, and the year-end employees, the number of enterprises, labor productivity, technical equipment rate and total profit as the explanatory variables, respectively, and draw the following data-related trend graph, so as to observe the possible functional relationship between the explanatory variables and the explained variables.

**Figure 3:**
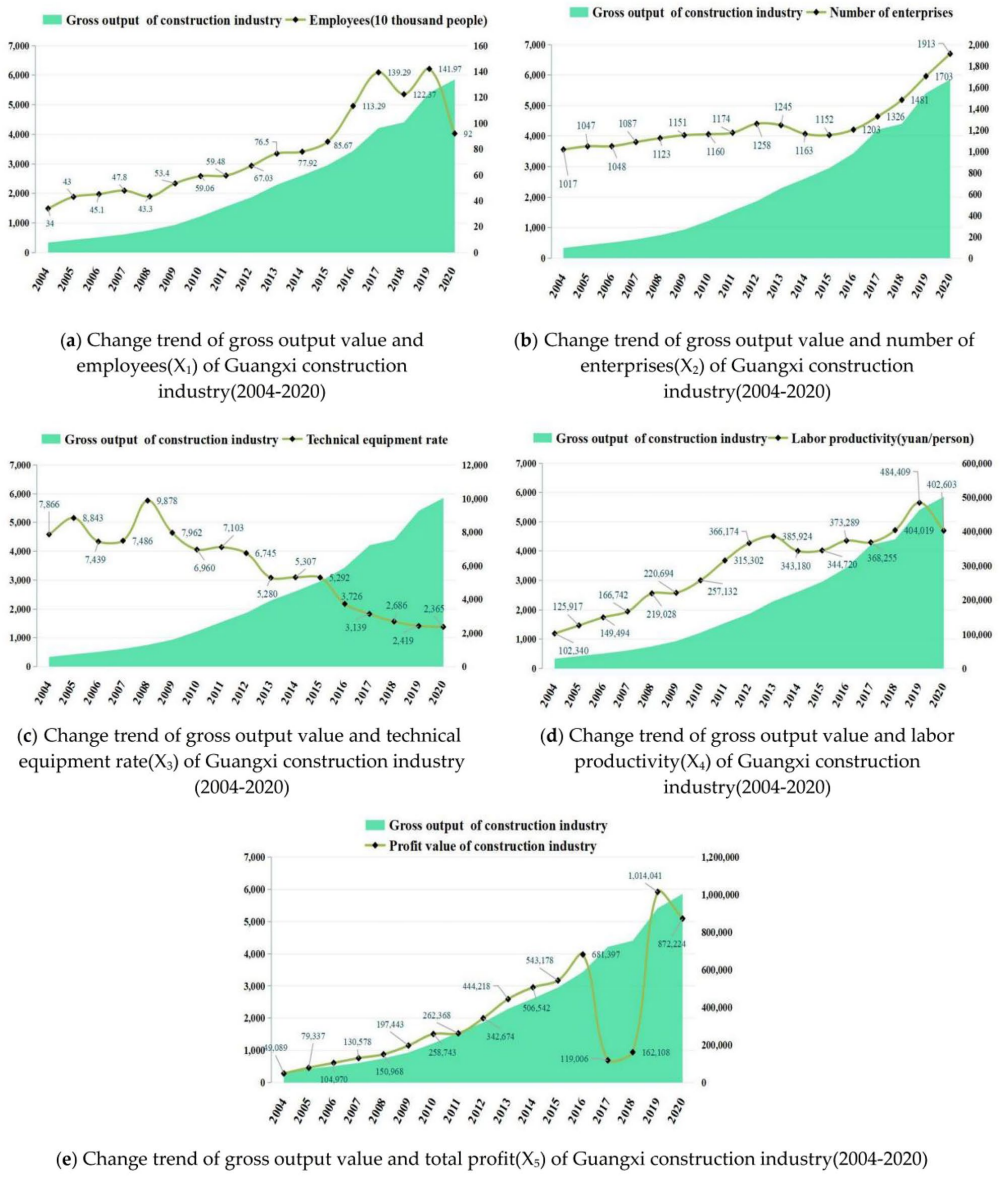
Change trend between gross output and another factors of of Guangxi construction industry from 2004 to 2020:(**a**) Change trend of gross output value and employees(X_1_) of Guangxi construction industry from 2004 to 2020;(**b**)Change trend of gross output value and number of enterprises(X_2_) of Guangxi construction industry from 2004 to 2020;(**c**)Change trend of gross output value and technical equipment rate(X_3_) of Guangxi construction industry from 2004 to 2020;(**d**)Change trend of gross output value and labor productivity(X_4_) of Guangxi construction industry from 2004 to 2020;(**e**)Change trend of gross output value and total profit(X_5_) of Guangxi construction industry from 2004 to 2020.

Figure 3 show the comparative relationship between the number of employees, the number of enterprises, labor productivity, technical equipment rate and total profit change with the total construction industry output value of Guangxi as the area graph respectively, similar to the conclusion of the previous analysis, except for the technical equipment rate index which shows the opposite development trend with the total construction industry output value, the change trend of other indexes is roughly the same as the change trend of the total construction industry output value, but the slope is different, from which the following conclusions can be drawn:

1. The total construction output value is correlated with the number of employees, enterprises, labor productivity, technical equipment rate and total profit at the end of the year.
2. The total construction output value is positively linearly related to the number of employees, enterprises, labor productivity and total profit at the end of the year, i.e., when the total construction output value increases, the labor productivity also increases; the total construction output value is inversely linearly related to the technical equipment rate, i.e., when the technical equipment rate increases, the total construction output value decreases.

The above qualitative analysis can provide the basis for the establishment and validation of the model below.

### Establishment of linear regression equation

According to the total factor productivity index system of Guangxi construction industry established in Table 1, year-end employees X1, number of enterprises X2, technical equipment rate X3, labor productivity X4, total profit of construction industry X5 are taken as variables observed on the gross output value of construction industry, thus establishing a multivariate linear equation:

2 $$Y_{i} = \beta_{0} + \beta_{1} X_{1i} + \beta_{2} X_{2i} + \beta_{3} X_{3i} + \beta_{4} X_{4i} + \beta_{5} X_{5i} + u_{i}$$

Expressed in matrix as:

3 $$Y = X\beta + u$$

And estimation is expressed as:

4 $$Y = X\hat{\beta } + \hat{u}$$

Due to:

5 $$\begin{gathered} X^{\prime}Y = \left[ {\begin{array}{*{20}c} 1 & 1 & \cdots & 1 \\ {X_{1,1} } & {X_{1,2} } & \cdots & {X_{1,17} } \\ {X_{2,1} } & {X_{2,2} } & \cdots & {X_{2,17} } \\ \vdots & \vdots & \vdots & \vdots \\ {X_{5,1} } & {X_{2,17} } & \cdots & {X_{5,17} } \\ \end{array} } \right]\left[ {\begin{array}{*{20}c} {Y_{1} } \\ {Y_{2} } \\ {Y_{3} } \\ \vdots \\ {Y_{17} } \\ \end{array} } \right] = \left[ {\begin{array}{*{20}c} 1 & 1 & \cdots & 1 \\ {34} & {43} & \cdots & {92} \\ {1017} & {1047} & \cdots & {1913} \\ \vdots & \vdots & \vdots & \vdots \\ {49089} & {79337} & \cdots & {872224} \\ \end{array} } \right] \times \left[ {\begin{array}{*{20}c} {333.59} \\ {425.21} \\ {512.83} \\ \vdots \\ {5853.24} \\ \end{array} } \right] \hfill \\ \hat{\beta } = \left( {X^{\prime}X} \right)^{ - 1} X^{\prime}Y \hfill \\ \end{gathered}$$

6 $$\hat{u} = Y - X\hat{\beta }$$

According to Formula 5 and 6,it can be calculated as formula 7:

7 $$\sigma^{2} = \frac{{e^{\prime}e}}{n - k - 1} = \frac{{\hat{u}^{\prime}\hat{u}}}{n - k - 1} = 9890743.44(n = 17,k = 5)$$

From this process, the estimator of the variance of random interference term is 9,890,743.44, and it is proved that the estimator is a linear unbiased estimator.

When some variables are observed or measured rather than operational, the estimation results based on these variables may be biasedbecause these variables may be correlated with the error term of the model, that is the endogeneity problem^35^.Due to the endogeneity of variables in linear regression equations, the instrumental variable method(IV) is often used in empirical studies in areas of management and economics to analyze the endogeneity of variables,and 2SLS is the most commonly used instrumental variable method^36,37^.The estimation method of 2SLS deals with endogeneity by using other exogenous variables in the model as instrumental variables, regressing all exogenous explanatory variables on the endogenous explanatory variables first, and the obtained estimates are then regressed on the explanatory variables together with the control variables, thus eliminating that part of the effect on the explanatory variables. This study adds Weak Instrument Diagnostics to the 2SLS regression analysis.

Through the calculation of Eviews software, Table 4 shows the regression analysis and calculation results of the total factor productivity index of Guangxi construction industry:

**Table 4:** Regression analysis of total factor productivity indicators of Guangxi construction industry.

Variable	Coefficient	Std.error	Prob.(2SLS)	Cragg-donald F-stat
Employees (10,000)	0.3918	0.3282	0.0056	55.987 (8.96)
Number of enterprises	0.0387	0.2919	0.0112	62.788 (8.96)
Technical equipment rate	− 0.2109	0.2463	0.0007	30.39 (8.96)
Labor productivity	1.1195	0.0855	0.0893	9.046 (8.96)
Total profit	0.0739	0.1919	0.0147	70.179 (8.96)
Constant C	14,4682.7	922,099.5	0.0001	–
R-squared	0.992642		0.917838	
Adjusted R-squared	0.990189		0.880492	
F-Statistic	4.7026		24.57645	
Prob.(F-Statistic)	0.0000		0.000013	
Durbin-Watson stat	1.9436		2.142885	

Stock-Yogo critical values in 15% level are in parentheses in the table,Cragg-Donald F-stat value not less than Stock-Yogo critical values in 15% level implies rejection of the original hypothesis of Weak Instrument Diagnostics.

The R-square value is significant, which proves that the model fit is better. The D.W. value is significant, which proves that there is no correlation effect between variable. At the same time ,the all P value of 2SLS are significant,and Cragg-Donald F-stat value not less than Stock-Yogo critical in 15% level, thus there is no weak instrumental variable, endogenous problem is tested and solved.The F-statistic and Prob. (F-Statistic) are significant. Generally speaking, the fitting results of the model are good.

Based on the data in Table 4, the model is estimated as:

8 $${\text{X}}_{6} = 144682.7 + 0.3918{\text{X}}_{1} + 0.0387{\text{X}}_{2} - 0.2109{\text{X}}_{3} + 1.1195{\text{X}}_{4} + 0.0739{\text{X}}_{5} + {\upsigma }^{2}$$

### Linear regression equation test

**Figure 4:**
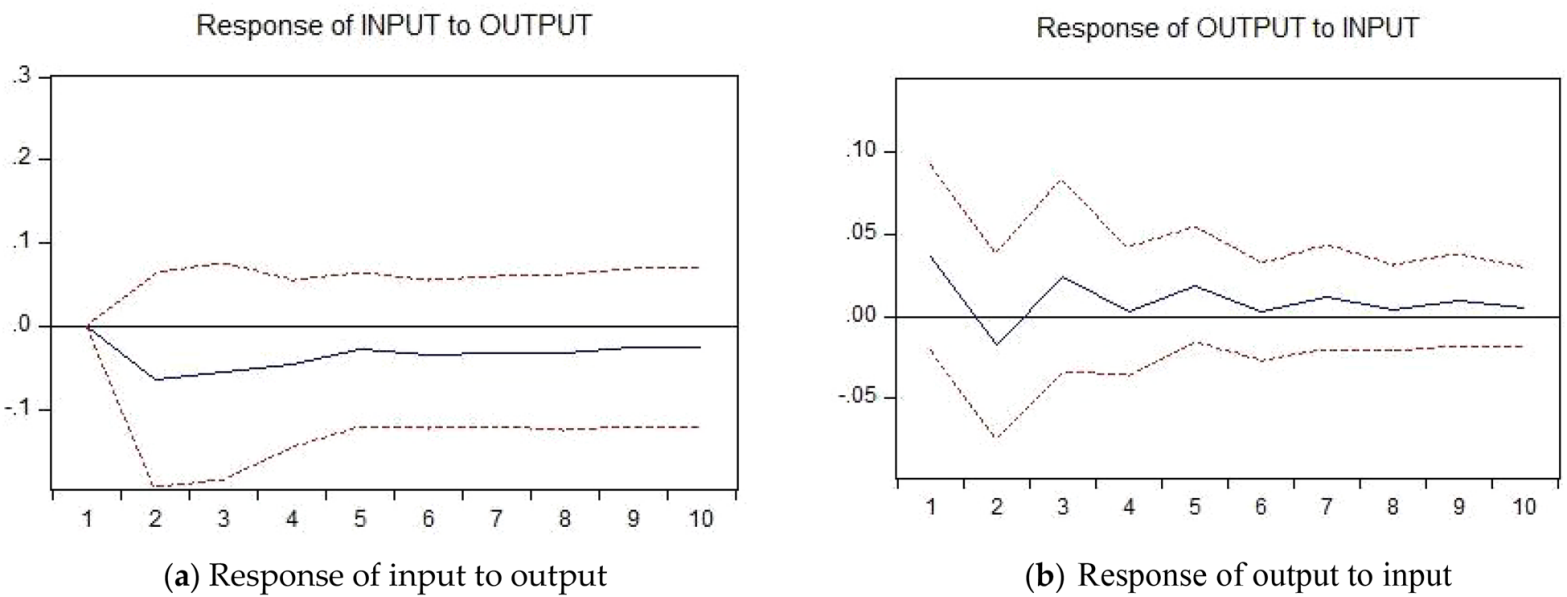
Impulse Response between input and output:(**a**)Response of input to output;(**b**)Response of output to input.

1. Economic significance test
  According to the estimation results of the model (formula 8), under the assumption that other variables keep unchange, the total output value of the construction industry will increase by 39.18 million yuan when the number of employees(X_1)_ increases by 10,000; the number of enterprises(X_2_) increases by one, the total output value of the construction industry will increase by 3.87 million yuan; the technical equipment rate(X_3_) increases by 1 yuan/person, the total output value of the construction industry will decrease by 2.109 billion yuan; the labor productivity increases by 1 yuan/person, the total output value of the construction industry will increase by 111.95 million yuan;the total profit increase by 10,000 yuan,the total output value of the construction industry will increase by 7.39 million yuan. And the linear relationship matched the pattern of Fig. 4, it passes the economic significance test.

(2) Fit test
  The R^2^ = 0.993 and Adjusted R^2^ = 0.990,of this linear regression equation proved the goodness of fit.

(3) F-test:
  For H_0_: β_1_ = β_2_ = β_3_ = 0 as the original hypothesis, the alternative hypothesis is H_1_:β_j_ ≠ 0,j = 1,2,…, k, given the significance level α = 0.05, and F_0.05_ (5,13) = 3.03 is found in the F distribution table. since F = 4.7026 > 3.03, the original hypothesis H_0_ should be rejected, indicating that the regression equation is significant, and That is, the number of employees (X_1_), number of enterprises (X_2_), technical equipment rate (X_3_), labor productivity (X_4_) and total profit(X_5_) have a significant effect on the gross value of construction industry (X_6_) with.

## Dynamic measurement of total factors production in construction industry of Guangxi province

The dynamic econometric analysis was conducted by establishing Vector Autoregression Model (VAR)^38^on the input–output indicators of all factors of production in Guangxi construction industry. There are four steps, firstly, unit root test is conducted to determine whether the panel data constitute a smooth series. Secondly, the Johansen cointegration test is conducted to test whether there is a cointegration relationship between the two input–output indicators^39^. In the third step, Granger Causality test was conducted to test the Granger causality relationship between the two indicators. The fourth step performs impulse response analysis between the two indicators to determine the degree of impact of shocks between the indicators^40,41^. Finally, the variance decomposition is applied to analyze the strength of the interaction between the inputs and outputs of the construction industry in Guangxi.

In order to maintain the consistency of calculation caliber, the original data are standardized, and the standardized data are shown in Table 5:

**Table 5:** Standardized data of indicators related to total factor productivity of Guangxi construction industry from 2004 to 2020.

Year	X_1_	X_2_	X_3_	X_4_	X_5_	X_6_
2004	0.00	0.00	0.00	0.73	0.00	0.00
2005	0.08	0.03	0.06	0.86	0.03	0.02
2006	0.10	0.03	0.12	0.68	0.06	0.03
2007	0.66	0.43	0.66	0.24	0.87	0.90
2008	0.09	0.12	0.31	1.00	0.11	0.08
2009	0.62	0.40	0.54	0.19	0.81	0.84
2010	0.23	0.16	0.41	0.61	0.22	0.16
2011	0.24	0.18	0.56	0.63	0.22	0.22
2012	0.31	0.27	0.69	0.58	0.30	0.28
2013	0.39	0.25	0.74	0.39	0.41	0.35
2014	0.41	0.16	0.63	0.39	0.47	0.41
2015	0.48	0.15	0.63	0.39	0.51	0.47
2016	0.73	0.21	0.71	0.18	0.66	0.56
2017	0.98	0.34	0.70	0.10	0.07	0.70
2018	0.82	0.52	0.79	0.04	0.12	0.74
2019	1.00	0.77	1.00	0.01	1.00	0.92
2020	0.54	1.00	0.79	0.00	0.85	1.00

### ADF test

To analyze whether the series is stable or not based on the time series, it can be tested. If a time series has stable mean, variance and self-covariance, then this series can be judged as stable, otherwise it should be judged as non-stationary series. If there is a non-stationary time series, the time division of the difference can be used to form a stationary series. Assuming that it is stable after passing the difference for d times, it is recorded as a single integer of order d (Integration) and is denoted as I (d). The unit root can be tested by ADF test (Augment Dickey-Fuller Test) to check whether the variables are stable, if the series is smooth, there is no unit root; otherwise, there is a unit root. Therefore, the H_0_ hypothesis of the ADF test is the existence of unit root, and if the significance test statistic obtained is less than three confidence levels (1%, 5%, 10%), it corresponds to having (99%, 95, 95%) certainty to reject the original hypothesis. The specific formula was calculated as follows^29,30^.

9 $$\Delta Y_{t} = \beta _{0} + \beta _{1} t + \delta Y_{{t - 1}} + \xi _{1} \Delta Y_{{t - 1}} + \cdots + \xi _{{p - 1}} \Delta Y_{{t - p + 1}} + u_{t}$$

where Y_t_ is the time series to be tested.

Use Eviews 10.0 software for calculation, and the order calculation results after automatic selection according to SIC criteria are shown in Table 6:

**Table 6:** ADF test results.

Varible	ADF inspection value	Critical value (1%)	Critical value (5%)	Critical value (10%)	Prob.*	Conclusion
INPUT	− 0.5311	− 3.959	− 3.081	− 2.681	0.8590	Unstable
OUTPUT	− 3.0130	− 3.959	− 3.081	− 2.681	0.0564	Stable at 10% level
X_1_	− 1.334	− 3.959	− 3.081	− 2.681	0.5850	Unstable
X_2_	0.944	− 3.959	− 3.081	− 2.681	0.9930	Unstable
X_3_	− 2.031	− 3.959	− 3.081	− 2.681	0.2717	Unstable
X_4_	− 0.311	− 4.004	− 3.099	− 2.690	0.9003	Unstable
X_5_	− 3.7006	− 3.920	− 3.066	− 2.673	0.0152	Stable at 1% level
X_6_	1.691	− 3.959	− 3.081	− 2.681	0.734	Unstable
**First order**						
INPUT	− 9.237	− 3.959	− 3.081	− 2.681	0.0000	Stable
OUTPUT	− 5.424	− 4.004	− 3.099	− 2.690	0.0023	Stable
X_1_	− 7.8645	− 3.959	− 3.081	− 2.681	0.0000	Stable
X_2_	− 5.5830	− 4.004	− 3.099	− 2.690	0.0045	Stable
X_3_	− 6.84	− 3.959	− 3.081	− 2.681	0.0001	Stable
X_4_	− 6.0639	− 4.004	− 3.099	− 2.690	0.0003	Stable
X_5_	− 6.6997	− 3.959	− 3.081	− 2.681	0.0001	Stable
X_6_	− 9.669	− 3.959	− 3.081	− 2.681	0.0000	Stable

The ADF test results show that the input index and output index of the total factor productivity of Guangxi, as well as the secondary factor indicators are not stable under the original conditions (with the output indicator stable at 10% significance level and the construction profit amount X_5_ stable at 1% significance level), which are not sufficient for the co-integration test, so the test is carried out after first-order difference. Therefore, the first-order difference is tested. All the first-order indicators and second-order indicators are smooth at 1%, 5%, and 10% significance levels after first-order differencing, which is a first-order single integer series and satisfies the precondition of cointegration test.

### Johansen cointegration test

Johansen co-integration test is used to test whether there is a long-term stable relationship between input and output indicators of Guangxi construction industry, which is calculated by Eviews software, and the test results are shown in Table 7:

**Table 7:** Johansen cointegration test results.

Hypothesis	Eigenvalue	Trace test			Maximum eigenvalue		
		Trace statistic	0.05Critical value	Prob.**	Max-eigen statistic	0.05Critical value	Prob.**
None*	0.6643	17.6268	15.4947	0.0235	16.3720	14.2646	0.0229
At most 1	0.0802	1.2548	3.8415	0.2626	1.2548	3.8415	0.2626

*denotes rejection of the hypothesis at the 0.05 level.

By comparing the statistics with the critical value of significance level (5%), there is a unique co-integration relationship between input and output indicators of Guangxi construction industry, which indicates that there is a long-term dynamic equilibrium relationship between input and output indicators of Guangxi construction industry.

### Granger causality test

Johansen cointegration test argues that there is a long-term dynamic equilibrium relationship between input and output indicators of the construction industry in Guangxi, and subsequently the Granger Causality Test^42^ can be conducted on these two variables, and the results are calculated using Eviews software as shown in Table 8.

**Table 8:** Granger causality test results.

Null hypothesis	F-Statistic	*P* value	Denote	Conclusion
Output does not Granger cause input	0.7675	0.4897	Accept	Output does not cause input
Input does not Granger cause output	1.1625	0.3516	Accept	Input does not cause output
∆Output does not Granger cause ∆Input	0.6537	0.4334	Accept	∆Output does not Granger cause ∆Input
∆Input does not Granger cause ∆Output	0.0439	0.8373	Accept	∆Input does not Granger cause ∆Output

∆ denotes the first order series of this data.

The Granger causality analysis test between input and output indicators found that input indicators and output indicators are not Granger causally related to each other, indicating that there is no statistically significant causal relationship between the two indicators. Taking the construction output indicator (X_6_) as the observed variable, the statistical causality between the components of the input indicator and this indicator is further analyzed, and the data are shown in Table 9:

**Table 9:** Granger causality test results about X_6_.

Null hypothesis	F-Statistic	*P* value	Denote	Conclusion
X_1_ does not granger cause X_6_	4.6894	0.0366	Reject	X_1_ does granger cause X_6_
X_6_ does not granger cause X_1_	0.9598	0.4156	Accept	X_6_ does not granger cause X_1_
X_2_ does not granger cause X_6_	7.7199	0.0094	Reject	X_2_ does granger cause X_6_
X_6_ does not granger cause X_2_	0.7294	0.5062	Accept	X_6_ does not granger cause X_2_
X_3_ does not granger cause X_6_	5.0716	0.0302	Reject	X_3_ does granger cause X_6_
X_6_ does not granger cause X_3_	1.8936	0.2007	Accept	X_6_ does not granger cause X_3_
X_4_ does not granger cause X_6_	1.9907	0.1872	Accept	X_4_ does not granger cause X_6_
X_6_ does not granger cause X_4_	0.6206	0.5571	Accept	X_6_ does not granger cause X_4_
X_5_ does not granger cause X_6_	4.1461	0.0408	Reject	X_5_ does granger cause X_6_
X_6_ does not granger cause X_5_	3.2189	0.0833	Accept	X_6_ does not granger cause X_5_

Granger causality analysis of each component of X1-X5 with the observation index X_6_ reveals that X_1_, X_2_, X_3_, and X_5_ are one-way Granger causality for X_6_, respectively, and X_4_ and X_6_ are not Granger causality for each other, indicating that there is a one-way coercive relationship between X_1_, the number of enterprises X_2_, labor productivity X_3_, and total profit X_5_ on construction output value at 5% significance level, and there is no statistical causality between technical equipment rate X_4_ and construction output value X_6_.

### Impulse response analysis

The impulse response analysis results shown as Fig. 4:

The impulse response analysis of the input and output indicators of Guangxi construction industry is carried out for 10 periods by Eviews software, and the images shown in Fig. 4a and Fig. 4b are obtained, where the solid line indicates the impulse response coefficient and the dashed line indicates the positive and negative two times standard deviation deviation bands. In general, the response of input indicators to output indicators fluctuates less and starts to stabilize in the later period after certain fluctuations in the first four periods,show as Fig. 4a, but the overall impulse response coefficient is negative; while the response of output indicators to input indicators fluctuates more, fluctuates frequently until 8 periods and stabilizes only after 9 periods,and in the first 2 periods, the output indicators show a negative impact on the input indicators, and then slowly rebound, reaching a peak of rebound in the fifth period, but the overall impact is still negative. Figure 4b shows that the input indicator has a negative impact on the output indicator in the first two periods, and then rebounded rapidly to a positive value in the third period, showing a fluctuating pattern from the third to the fifth period, but the impulse response coefficient is mostly in the positive direction, and only drops steeply to a negative value in the second period.

### Variance decomposition

The impulse response analysis in the previous step can reveal the relationship formed by the factors impacting each other, and in order to further analyze the independence of the factors themselves and the extent to which they are influenced by other variables, which can reflect the relative relationship between the endogenous variables of the model, the analysis can be performed by means of variance decomposition^43,44^. The results of the variance decomposition are shown in Table 10:

**Table 10:** Variance decomposition results.

No	INPUT			OUTPUT		
	S.E	INPUT/%	OUTPUT/%	S.E	INPUT/%	OUTPUT/%
1	0.2165	100	0.0000	0.1120	10.3647	89.6353
2	0.2293	91.9771	8.0229	0.1158	12.0246	87.9754
3	0.2857	91.0914	8.9086	0.1226	14.7229	85.2771
4	0.2955	89.3414	10.6586	0.1252	14.1672	85.8328
5	0.3202	90.1428	9.8572	0.1266	16.0924	83.9073
6	0.3281	89.5450	10.4555	0.1267	16.1089	83.8911
7	0.3426	89.5731	10.4269	0.1273	16.8286	83.1714
8	0.3495	89.1603	10.8397	0.1275	16.8967	83.1033
9	0.3586	89.1651	10.8350	0.1279	17.3511	82.6489
10	0.3638	88.9929	11.0071	0.1280	17.4702	82.5289

From the variance decomposition data, it can be seen that for the construction industry input indicators, they are mainly influenced by themselves, with a probability level of 100% in the first period and a probability level of around 90% from the second to the tenth period, with a peak impact on the output indicator level at 11%. For the construction industry output indicators are also influenced by themselves, from the first period to the tenth period are in the range of 80%–90%, the level of output indicators have a certain impact impact on them, the peak in the tenth period appeared at 17.47%. It can be seen that the output indicators have a stronger ability to explain the city input indicators than the input indicators to the output indicators.

## Conclusions and suggestion

### Conclusions

Through the measurement of the total factor productivity of Guangxi's construction industry, as well as the dynamic econometric analysis within the output indicators and the component indicators of the output indicators, the following conclusions can be obtained:

1. The TFP of Guangxi construction industry has been at a low level except for 2017 and 2018 when there was a significant increase, and the overall trend has been lower year by year, which deserves attention.This is consistent with the conclusion of Xiahou et al. for the TFP of all provinces in China^45^.

(2) The gross product of Guangxi construction industry has a certain coupling effect with the number of employees, enterprises, labor productivity and total profit of Guangxi construction industry, showing a positive correlation, and the technical equipment rate shows a negative correlation with the gross product of Guangxi construction industry, indicating that the improvement of the gross product of Guangxi construction industry does not drive the improvement of the technical equipment rate. As the traditional industry of construction industry, the level of technical equipment rate should be located at a high level, which means that the technical force and hardware facilities have sufficient guarantee, while the gross value of Guangxi construction industry does not lead to the improvement of technical equipment rate, which means that Guangxi construction industry is still at a lower level of technology.

(3) There is a dynamic equilibrium relationship between input and output indicators of total factor productivity of Guangxi construction industry, but the mutual impact effect of the two indicators is different, the impact of output indicators on input indicators fluctuates less and the impulse response coefficient is negative, which means that the positive driving effect of output indicators on input indicators is not obvious; the impact of input indicators on output indicators fluctuates more and the impulse response coefficient is mostly positive except for occasional negative values, which means that the impact of input indicators on output indicators is greater and the positive impact is more, so we can focus on controlling the component indicators of input indicators to improve the level of Guangxi construction industry.

The conclusion of the research shows that the overall economic and technological level of Guangxi's construction industry is at a low level in China, and has not achieved breakthrough growth for many years. However, through the correlation and dynamic econometric analysis about TFP’s input and output indicators of Guangxi, it is found that increasing the input indicators can significantly positively affect the output indicators. Therefore, it is necessary to pay attention to and give full play to the pulling effect of input index on output index.This is consistent with the research conclusions of Li Zhan et al.^46^, and enriches the research results of Ye Gui et al.^47^, and replenish the provincial data of China’s contruction industry.

### Suggestion

The recommendations of this study to improve the level of Guangxi's construction industry are as follows:

1. The level of employees and technology in Guangxi is still in the middle or even backward of the 31 provinces and cities in China, and reflects the strong correlation between the construction industry and regional economic development. Therefore, it is necessary to increase investment, expand scale, optimize structure and enhance production capacity. Guangxi construction enterprises are still in the investment-driven stage, the expansion of enterprise production scale, need to increase investment. Increasing the investment of capital, talent, technology and equipment and other important production factors, adjusting and optimizing the enterprise management structure to enhance the production capacity of enterprises and realize the connotation of expanding reproduction is very important. Relevant government departments should focus on improving technical equipment capacity and increase investment in science and technology innovation, and strengthen the importance and support of construction technology in terms of policy, management, funds and talents, etc.

(2) Acccording to the situation of Guangxi’s construction industry , the sustainable development planning must be made. Promoting the layout of key technology, the layout of design and R&D bases, the layout of parts production bases, etc., putting forward the modernization of the construction industry, and strengthening the cultivation and promotion of new construction enterprises and new construction technologies, increasing the market competitiveness of enterprises through the upgrading of management structure and technology, so as the profit space and sustainable development power of Guangxi construction industry will improve.

This study has some limitations due to data statistics and indicator screening, and the types of indicators can be expanded to carry out more extensive research in the future. The research path and research methods in this paper can also be used in the analysis of other industries to provide ideas for the management improvement of different industries.

## Data Availability

All data generated or analysed during this study are included in this published article. These data can be found in China Statistical Yearbook (http://www.stats.gov.cn/english/) and Guangxi Statistical Yearbook (http://tjj.gxzf.gov.cn/tjsj/tjnj/).
